# Identification and profiling of narrow-leafed lupin (*Lupinus angustifolius*) microRNAs during seed development

**DOI:** 10.1186/s12864-019-5521-8

**Published:** 2019-02-14

**Authors:** Kathleen DeBoer, Su Melser, Jana Sperschneider, Lars G. Kamphuis, Gagan Garg, Ling-Ling Gao, Karen Frick, Karam B. Singh

**Affiliations:** 1 The UWA Institute of Agriculture, University of Western Australia, Crawley, WA 6009 Australia; 2 CSIRO Agriculture and Food, Private Bag 5, Wembley, WA 6913 Australia; 3 Centre for Genomics, Metabolomics and Bioinformatics (CGMB), The Australian National University, Canberra, ACT 2601 Australia; 4 Curtin University, Centre for Crop and Disease Management, Department of Environment and Agriculture, Bentley, WA 6102 Australia; 5 The School of Plant Biology, University of Western Australia, Crawley, WA 6009 Australia; 6 Present address: INSERM U1215, Neurocentre Magendie, Bordeaux, France

**Keywords:** Gene silencing, Legume, Lupin, microRNAs, Seed development, Small RNAs

## Abstract

**Background:**

Whilst information regarding small RNAs within agricultural crops is increasing, the miRNA composition of the nutritionally valuable pulse narrow-leafed lupin (*Lupinus angustifolius*) remains unknown.

**Results:**

By conducting a genome- and transcriptome-wide survey we identified 7 *Dicer-like* and 16 *Argonaute* narrow-leafed lupin genes, which were highly homologous to their legume counterparts. We identified 43 conserved miRNAs belonging to 16 families, and 13 novel narrow-leafed lupin-specific miRNAs using high-throughput sequencing of small RNAs from foliar and root and five seed development stages. We observed up-regulation of members of the miRNA families miR167, miR399, miR156, miR319 and miR164 in narrow-leafed lupin seeds, and confirmed expression of miR156, miR166, miR164, miR1507 and miR396 using quantitative RT-PCR during five narrow-leafed lupin seed development stages. We identified potential targets for the conserved and novel miRNAs and were able to validate targets of miR399 and miR159 using 5′ RLM-RACE. The conserved miRNAs are predicted to predominately target transcription factors and 93% of the conserved miRNAs originate from intergenic regions. In contrast, only 43% of the novel miRNAs originate from intergenic regions and their predicted targets were more functionally diverse.

**Conclusion:**

This study provides important insights into the miRNA gene regulatory networks during narrow-leafed lupin seed development.

**Electronic supplementary material:**

The online version of this article (10.1186/s12864-019-5521-8) contains supplementary material, which is available to authorized users.

## Background

Grain legume crops, such as chickpea (*Cicer arieratum*), common bean (*Phaseolus vulgaris*), faba bean (*Vicia faba*), lentil (*Lens culinaris*), pea (*Pisum sativum*) and narrow-leafed lupin (*Lupinus angustifolius*) are important in crop rotations due to their ability to fix their own nitrogen, therefore reducing the reliance on commercial fertilizers and providing a break in the disease cycles in cereal and oilseed crops [1]. Grain legumes, also known as pulses, are of particular importance in vegetarian diets as an important source of protein and micronutrients while also being low in fats. Narrow-leafed lupin for human consumption, has a number of attractive nutritional attributes relating to their high protein content, high dietary fibre and negligible starch levels [2]. Recent studies have also demonstrated a number of human health benefits associated with consumption of lupin grains, particularly in the areas of cardiovascular disease, diabetes and obesity [3–5]. Despite the attractive properties of the lupin grain as a food source, this legume of the Genistoid clade has only recently been domesticated [6], with current elite cultivars having a very narrow-genetic base [7]. Thus there is a considerable potential to improve the quality of the lupin grain and its yield.

A number of genetic and genomic resources have recently been developed for narrow-leafed lupin to accelerate crop improvement. These include the generation of various BAC libraries [8, 9], transcriptome datasets [10, 11], a comprehensive reference genome [12], various re-sequenced cultivars [13], large sets of molecular markers and two dense genetic maps [11, 14, 15] as well as BAC-FISH cytogenetic markers assigning each linkage group to a chromosome [16]. The generation of in depth transcriptome datasets for a number of different tissue types [10, 11], combined with a quality reference genome assembly [12], enables the investigation into the small RNA complement of narrow-leafed lupin.

Plants contain several classes of endogenous small RNAs, the most abundant being small interfering RNAs (siRNAs) produced from double stranded RNA precursors, which depending upon their biogenesis, can be further classified into heterochromatic siRNAs, secondary siRNAs and natural antisense transcripts siRNAs (nat-siRNAs) [17]. MicroRNAs are another abundant and ubiquitous class of small RNAs, and unlike siRNAs are derived from single stranded RNA transcript precursors that form imperfect hairpin-like structures [17]. In plants, miRNAs are involved in the post-transcriptional regulation of diverse aspects of growth and seed development, as well as stress tolerance [18]. In Arabidopsis, miR156 prevents over-accumulation of SPL10 and SPL11 transcription factors that may otherwise lead to abnormal embryo development [19]. Negative regulation of ARF10 by miR160 plays a critical role in seed germination [20]. Specific loss of miR166 can cause the ectopic expression of seed maturation genes in Arabidopsis [21] and they may play an important role in soybean seed development [22]. miR164 may play a role in normal embryonic development by degrading CUC1 and CUC2 mRNAs [23]. Thus, further focus on seed expression of miRNAs should help understand the roles of miRNAs in seed development.

The miRNA genes are initially transcribed by RNA polymerase II as “long” single stranded primary transcripts (pri-miRNA) which form imperfect hairpin stem-loop structures [24, 25]. These hairpin stem-loop structures are processed by DICER like-1 (DCL1) RNase III endonucleases which function in concert with the accessory proteins DOUBLE-STRANDED RNA-BINDING1 (DRB1; also known as HYPONASTIC LEAVES1) and SERRATE (SE), to produce precursor-miRNA (pre-miRNAs) sequences [26–28]. The precursor miRNAs undergo additional cleavage by DCL1 to release the ~ 21 bp miRNA (guide)/miRNA*(passenger) duplex, which is stabilised by 2’-O-methylation by HUA ENHANCER 1 (HEN1) [29]. Following export from the nucleus, the miRNA duplex is bound by ARGONAUTE1 (AGO1), forming the RNA induced silencing complex (RISC) which directs cleavage of target mRNA sequences with a high degree of complementarity to the miRNA-guide, via the endonuclease activity of AGO1 [30–32]. Some plant miRNAs can also direct translational inhibition of their targets by preventing the recruitment of ribosomes during translation initiation or blocking ribosome movement during translation elongation [33, 34]. MiRNAs can also function as long distant signalling molecules and have been found within the phloem [35, 36].

The number of plant miRNAs deposited in public databases such as miRBase has increased rapidly over the last two decades [37]. Over 20 miRNA families are conserved amongst the angiosperms with a large number of these targeting genes encoding transcription factors, which regulate important aspects of plant growth and development [37, 38]. Plants also contain a number of lineage specific miRNAs. New miRNA genes can be formed via a number of mechanisms including the duplication of existing miRNA genes, the inverted duplication of protein coding genes or through transposable elements [39]. Linage specific miRNAs are generally found as single copy genes, have a high birth-death rate, low expression and have few if any targets [40]. As such, most new linage specific miRNAs are often considered to be ‘transient’ or evolutionary neutral [41].

A number of miRNA identification studies have been undertaken in legumes, with the model legume *Medicago truncatula* and soybean (*Glycine max*) being the most comprehensively studied. For these two legumes species, miRNAs have been identified in different tissue types, during nodulation and mycorrhization and under various abiotic stress conditions [42, 43]*.* For instance, miR1507 has been identified only in legume species so far, and may play a key role in nodulation [43]. MiRNA identification studies have also been undertaken in the common bean (*P. vulgaris)* [44], peanut (*Arachis hypogaea*) [45] and mung bean (Vigna mungo) [46]. Only two miRNA studies have been undertaken on species within the *Lupinus* genus, with both of these studies focused on white lupin (*Lupinus albus*) [47, 48]. In one study, 383 small RNA sequences were isolated and sequenced from the phloem exudate, with 17 of these sequences exhibiting homology to 7 miRNA families previously deposited on miRBase [47]. The other study examined the global expression of miRNAs under phosphate deficiency in white lupin plants using microarray analysis and identified 30 families of miRNAs, with several showing tissue specificity [48].

In this report we first examined how well conserved the machinery for generating small RNAs was in narrow-leafed lupin compared to other legumes, given narrow-leafed lupin’s position in legume phylogeny within the Genistoid clade, which is quite distant from other well characterised legumes [12, 14]. For this analysis, we focused on the *Dicer-like* and *Argonaute* genes, identifying members of these gene families in the narrow-leafed lupin genome, examining their expression profiles and also their phylogeny relative to other *Dicer-like* and *Argonaute* gene members in legumes. We then undertook a comprehensive analysis of the small RNA profile of *L. angustifolius* cultivar Tanjil, with a focus on seed developmental stages. We describe the identification of conserved and novel miRNAs as well as the in silico identification of their putative targets. We confirmed the small RNAseq expression profiles using Quantitative PCR and validated a subset of putative targets using 5′ RLM-RACE analysis. These findings expand the list of annotated miRNAs for legumes and provides important insights into the miRNA gene regulatory networks during narrow-leafed lupin seed development and will lay the foundations to improve the quality of the lupin grain in the future.

## Methods

### Plant material

*L. angustifolius* (cultivar Tanjil) seeds were obtained from the Department of Primary Industries and Regional Development (DPIRD) of Western Australia who maintain the Australian lupin collection. Seeds were germinated and grown in temperature controlled growth cabinets at 22 °C day for 16 h and 20 °C night for 8 h. Once plants started to flower and set seed on the main stem, foliar tissue (stems and leaves) was harvested, as well as five different seed developmental stages: 4–8 DAA (days after anthesis), 9–20 DAA, 21–26 DAA, 27–32 DAA, and 39–44 DAA for subsequent RNA extraction.

### Identification of argonaute and dicer-like genes in the narrow-leafed lupin genome

The lupin genome assembly (version 1.0; [12]), was interrogated with sequences of *Dicer-like* (DCL1–4) and *Argonaute* genes (AGO1–10) from *Arabidopsis thaliana* and their predicted homologs in *Medicago truncatula*, *L. japonicus* and *Glycine max* described in [42]. As an additional line of evidence, Hidden Markov Model (HMM) analysis using conserved PFam and InterPro term domains were used to search for DCL and AGO genes encoded in the annotated gene set of the narrow-leafed lupin genome. Phylogenetic trees for the DCL and AGO genes were generated using the ETE 3 software with the standard_raxml workflow [49]. This used Clustal Omega as the aligner [50] and RAxML for phylogenetic tree prediction [51].

### RNA isolation, small RNA isolation and generation of small RNA sequencing libraries

Total RNA was isolated from the foliar and seed tissue using Trizol reagent (Invitrogen, Carlsbad, CA) as described by [11]. The generation of TruSeq smallRNA libraries (Illumina, San Diego, CA) was performed according to manufacturer’s recommendations using the TruSeq small RNA library sample preparation kit with an input amount of 1 μg total RNA and sequenced using an Illumina MiSeq. The raw data for each of the libraries were deposited in GenBank under BioProject ID: PRJNA299755.

### Computational analysis of small RNAs, miRNA identification and differential expression analysis

Adapters were trimmed using cutadapt (−m18 –M28 -q30 –trim-n –discard-untrimmed) [52]. Untrimmed reads, reads shorter than 18 nts or reads larger than 28 nts were discarded and flanking N bases were removed from each read [52]. Additional filtering was performed by removal of reads that mapped to rRNA, tRNA, and snoRNA using bowtie 1.1.2 [53].

For miRNA identification, we used the ShortStack 3.8.5 software [54] on the clean sRNA reads. We further filtered the predicted sRNA clusters to include only those where > = 80% of reads are within 20–24 nts of length. This is the recommended procedure in ShortStack to avoid degradation products. We also included only sRNA clusters with at least 2 reads per million (RPM). We used the read counts returned by ShortStack for all predicted sRNA clusters and used edgeR [55] to assess which are differentially expressed at any of the infection stages versus germinated spores (FDR < 0.05, fold change > 2).

The conserved and novel miRNAs were aligned to the cv. Tanjil reference genome assembly with ShortStack [54] and their scaffold coordinates used to place the miRNAs onto the narrow-leafed lupin pseudochromosomes and visualised using the MapChart v2.2 software [56]. The miRNA star sequences/secondary structures as predicted by ShortStack are provided in Additional file 1.

### Prediction of miRNAs target genes

MiRNA targets were identified using the psRNATarget software (2011 release) [55] against the narrow-leafed lupin transcriptome datasets [11]. Targets were predicted using the default parameters, except the maximum expectation cut off score was set to 2.5.

### Real-time quantitative RT-PCR of narrow-leafed lupin miRNAs and their target genes

Prior to RNA isolation, 100 mg of tissue was spiked with 100 nM of a random RNA sequence (UUAAGGCACGCGGUGAAUGCCAGCAGUGGC). The miRNA was isolated by using the PureLink® miRNA Isolation Kit (Qiagen) according to the manufacturer’s instruction. The small RNA fraction was polyadenylated using poly(A) polymerase (NEB), and reverse transcribed using superscript III (Thermo Fisher Scientific) and a poly(T) adaptor primer as described in [57]. qRT-PCR was performed using SsoFast EvaGreen Supermix (Bio-Rad) in 10 μL reactions on a CFX384 (Bio-Rad) system. Thermocycling conditions for each miRNA was determined experimentally. In general, thermocycling conditions were as followed: 95 °C for 2.30 min (1 cycle); 95 °C for 15 s, 60 °C for 30 s, 72 °C for 30 s (40 cycles), followed by a dissociation curve analysis of 65–95 °C at 0.5 °C per cycle. Quantification was performed using the 2^-∆CT^ method, with miRNA expression normalised relative to the random RNA sequence. The primer sequences used for qPCR analysis can be found in Additional file 2: Table S1.

### RLM- 5’ race

A modified RLM- 5’ RACE was performed essentially as described in [58, 59]. Briefly, total RNA was ligated to an RNA adaptor (CGACUGGAGCACGAGGACACUGACAUGGACUGAAGGAGUAGAAA), and reverse transcribed using superscript III (Thermo Fisher Scientific) using a gene specific primer. PCR amplification was undertaken using primers designed within the adaptor sequence and gene specific primers located approximately 400-500 bp downstream from the putative cleavage site (Additional file 1: Table S1). A nested PCR reaction was performed and PCR products corresponding to the predicted size sub-cloned into either pGEM Teasy TA vector or TOPO – TA vector and transformed into *E. coli* with plasmid from 10 to 15 independent colonies sequenced.

## Results

### Identification of Argonaute orthologs within the lupin genome

As the first step in studying the miRNA composition of narrow-leafed lupin we analysed some of the components of the cellular machinery involved in generating the miRNAs, namely the *Argonaute* and the *Dicer-like* gene complement within narrow-leafed lupin. The lupin genome assembly was interrogated with sequences of *Dicer-like* (*DCL1–4*) and *Argonaute* genes (*AGO1–10*) from *Arabidopsis thaliana* and their predicted homologs in *M. truncatula*, *L. japonicus* and *G. max* [42, 60, 61]. As an additional line of evidence, Hidden Markov Model (HMM) analysis using conserved Pfam domains and InterPro terms were used to search for *DCL* and *AGO* genes encoded in the annotated gene set of the narrow-leafed lupin genome.

**Fig. 1:**
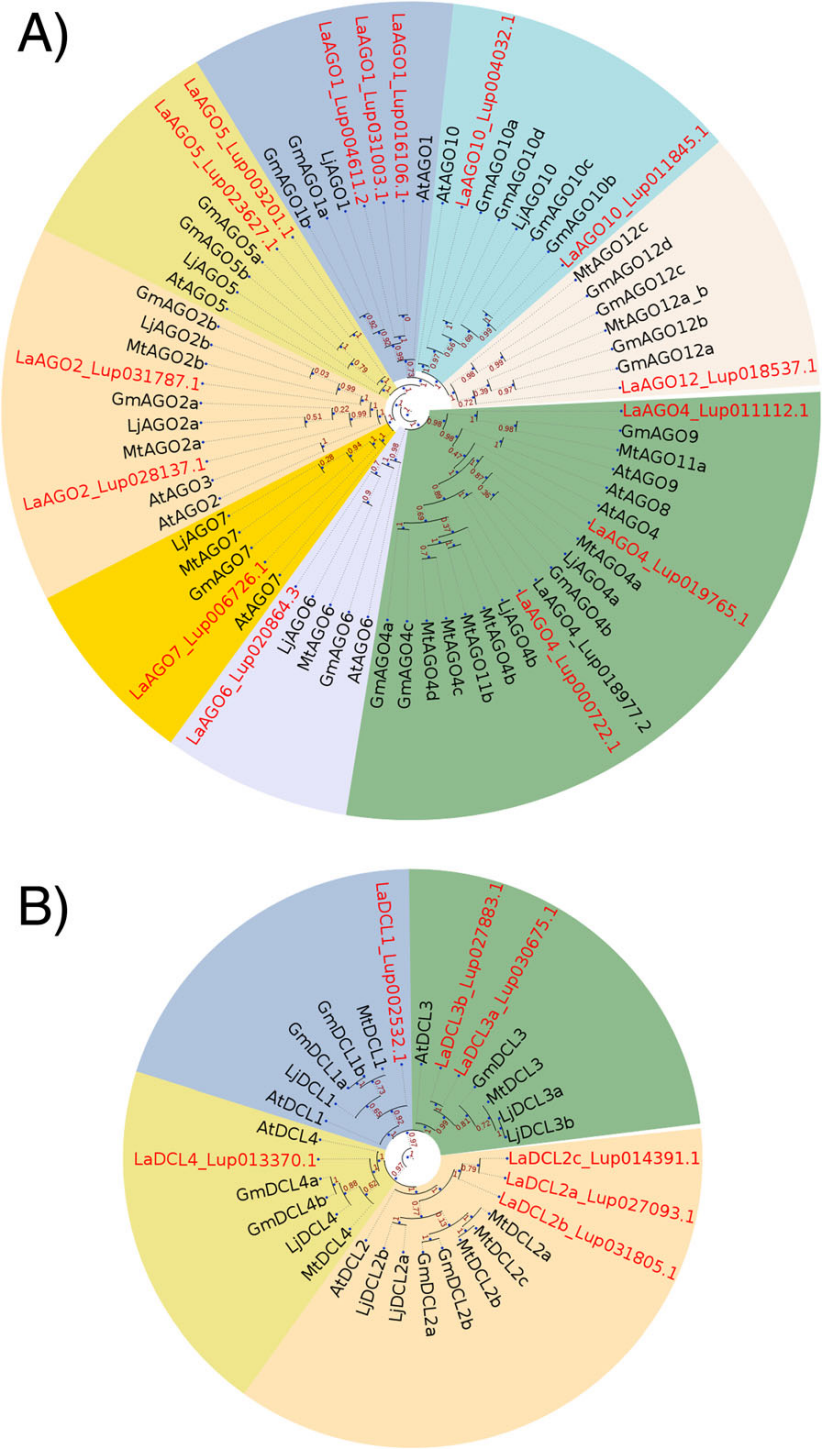
Phylogenetic analysis of narrow-leafed lupin orthologs of *AGO* and *DCL.* **a** *AGO-like* gene phylogeny (**b**) *DCL-like* gene phylogeny. Trees were predicted using the ETE 3 software [49]

A total of 16 *Argonaute* (*AGO*) genes were identified in the narrow-leafed lupin genome (Fig. 1a; Additional file 2: Table S2). Plant AGO proteins can be classified into three distinct clades; clade I (AGO1*/*AGO5*/*AGO10); clade II (AGO2/AGO3/AGO7); and clade III (AGO4*/*AGO6*/*AGO8/AGO9). An additional subclade is present within the grasses which contains the AGO18 proteins [61]. Phylogenetic analysis revealed strong homology of the narrow-leafed lupin *AGO* genes to those of other legumes (*G. max, M. truncatula* and *L. japonicas*) (Fig. 1a). We identified three *AGO1-like* and *AGO10-like* genes, two *AGO5-like* and *AGO2-like* genes and single members for *AGO6* and *AGO7.* Finally four *AGO4-like* members were identified in the narrow-leafed lupin genome. AGO4 associates with 24 nt small RNAs, mediating DNA methylation via the RNA-directed DNA methylation (RdDM) pathway in other plants species [62].

Changes in expression profiles following gene duplication events can facilitate functional diversification, therefore we examined the expression profiles of the *AGO* genes using the transcriptome datasets of five narrow-leafed lupin tissue types (root, stem, leaf, flower and seed) previously described [11]. Each of the *AGO* genes were expressed within the tissues examined, with a subset of *LaAGO* genes highly expressed within the seed (Additional file 3: Figure S1A). In particular, three members of the narrow-leafed lupin *LaAGO4* family (*LaAGO4a, LaAGO4c* and *LaAGO4d*) showed notably higher expression levels in the seed tissue compared to the other tissue types. *LaAGO1b* also exhibited notably higher expression level in the seed compared to other tissue types. This may be indicative of a specialised function for these AGO4 and AGO1 orthologues in narrow-leafed lupin seed development.

### Identification of dicer-like orthologs within the lupin genome

We also examined the *Dicer-like* gene complement within narrow-leafed lupin. Plants contain four *Dicer-like* (*DCL*) families (*DCL1–4*) [63]. Here we identified seven *DCL* genes in the narrow-leafed lupin genome including three gene members for *DCL2*, two members for *DCL3* and a single gene member for *DCL1* and *DCL4*, all of which exhibited close homology to other legume *DCL* genes (Fig. 1a; Additional file 3: Table S2). We also examined the expression profile of the narrow-leafed lupin *DCL* genes using the transcriptome datasets described above (Additional file 4: Figure S1B). *LaDCL1* and *LaDCL4* were highly expressed in all tissue types examined. *LaDCL2a, LaDCL2b, LaDCL2c* and *LaDCL3a* were also highly expressed, though differences in expression levels were observed between tissue types. *LaDCL2a*, *LaDCL2c* and *LaDCL3a* show highest expression levels in the seed tissue compared to the other tissue types. We did not detect *LaDCL3b* expression in any of the five different tissue types examined. Furthermore, *LaDCL3b* is also lacking the first 217 amino acids when compared with *LaDCL3a*, and thus may be a pseudogene. In *Arabidopsis*, DCL3 produces 24 nt siRNAs that function in DNA methylation [64].

### Sequencing of narrow-leafed lupin small RNAs

To compile a comprehensive list of narrow-leafed lupin miRNA sequences, with a focus on seeds, we generated small RNA sequencing libraries for foliar tissue (leaf and stem), root tissue and five different seed developmental stages; 4–8 days DAA, 9–20 DAA, 21–26 DAA, 27–32 DAA, and 39–44 DAA. We chose these stages as they involve discrete steps of lupin seed development as described in [65]. Briefly, embryogenesis occurs during the early stages of seed development during which the zygote differentiates into the embryo proper. By approximately 9–20 DAA the seed has transitioned into the maturation phase, during which cell division has decreased and storage proteins have begun to accumulate [66]. Once the seed has reached its physiological maturity by approximately 39–44 DAA, water is rapidly lost and the seed enters into a state of dormancy [65, 66]. In addition to the seed developmental stages we generated small RNA libraries for stem, leaf and root tissue to represent vegetative tissue types, which would allow us to identify seed specific miRNAs.

**Fig. 2:**
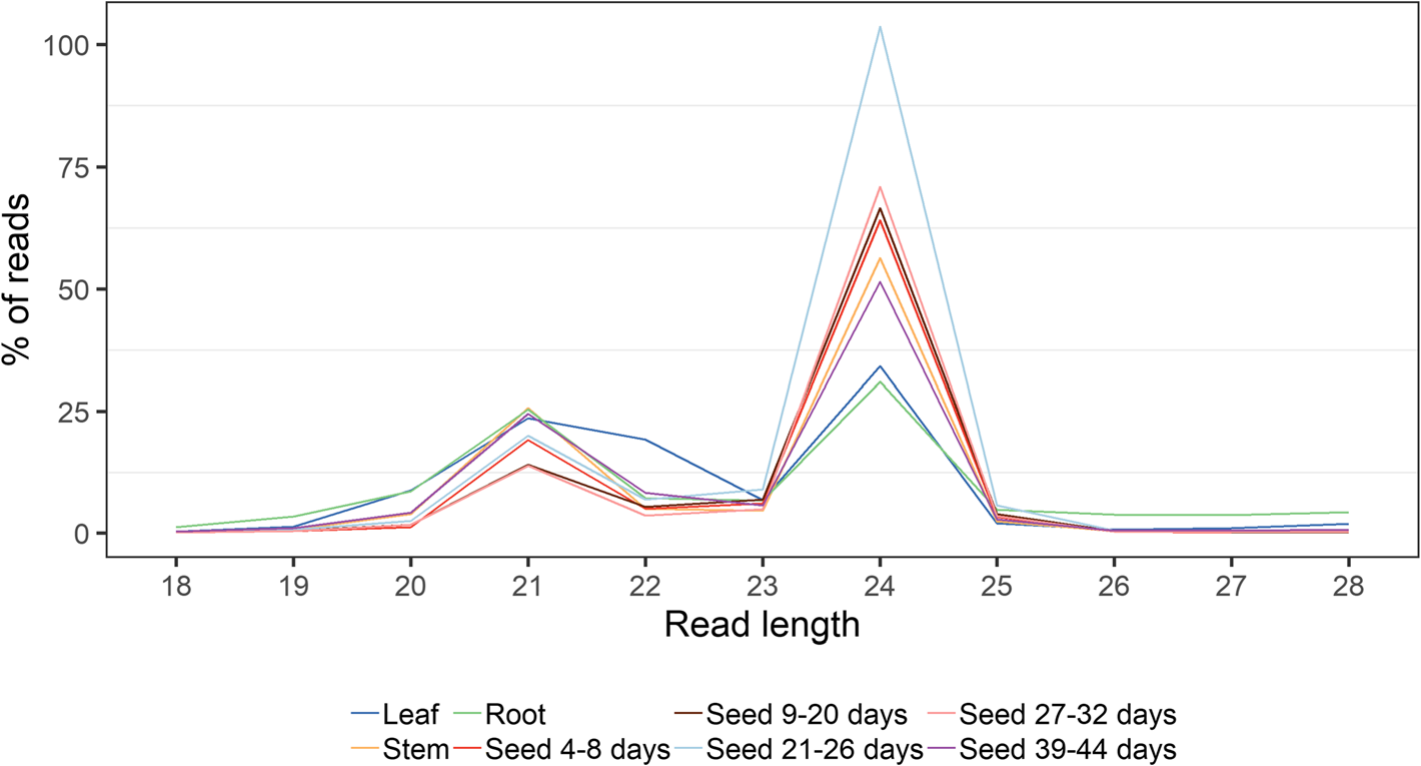
Read length distributions in the leaf, stem, root and seed samples. Peaks occur at 21 nt and 24 nt in all samples, as well as at 22 nt in the leaf sample

Libraries were generated and sequenced on an Illumina MiSeq. Reads were adapter-trimmed and those that mapped to rRNA, tRNA or snoRNA were removed. Following filtering, a total of 5,063,406 reads ranging in size from 18 to 28 nt remained. The small RNA reads show peaks at 21 nt and 24 nt, characteristic for plant small RNAs (Fig. 2). Interestingly, we observed a strong presence of 22 nt small RNAs in the leaf sample. In plants, phased small interfering RNAs (phasiRNAs) are produced in a one-hit or two-hit model, where mRNA targets are cleaved by a 22 nt miRNA. In legumes, phasiRNAs are often produced from coding disease resistance genes and appear to be beneficial for plant-microbial interactions and plant immunity [67].

### Identification of conserved miRNA sequence within the small RNA datasets

Identification of miRNA and small RNA sequences was undertaken using the ShortStack software [54]. Briefly, ShortStack performs alignment of small RNA-seq data as well as annotation and quantification of small RNA-producing genes. ShortStack predicts miRNAs using a range of stringent criteria, in line with [68]. Using ShortStack, we identified a total of 56 miRNAs within the lupin small RNA datasets, of which 43 were classified into 16 known miRNA families. 13 miRNAs did not have a hit to a known miRNA and were thus classified as novel miRNAs (Table 1). The predicted miRNAs were predominately 21 nt in length (73.2%), with lower proportions of 20 nt (8.9%) and 22 nt (12.5%) miRNAs present within the datasets. Furthermore, 75% of the predicted miRNA sequences contained a 5′ uridine nucleotide bias in accordance with observations in *A. thaliana* where miRNAs that associate with AGO1 predominately have a 5′ uridine nucleotide [69].

**Table 1:** Predicted miRNAs in lupin using ShortStack [54]

miRNA	miRNA Family	Genomic location	Sequence
Lan-miR-1507a	miR1507	Scaffold_24_1:1525561–1,525,719	CCUCGUUCCAUACAUCAUCUAG
Lan-miR-1507b		Scaffold_56:1091677–1,091,814	UCUCACUCCAUACAUCGUCUCG
Lan-miR-1511	miR1511	Scaffold_14_1:1672129–1,672,224	AACCAGGCUCUGAUACCAUGA
Lan-miR-156a-1	miR156	Scaffold_23:1019283–1,019,373	UGACAGAAGAGAGUGAGCAC
Lan-miR-156a-2		Scaffold_24_1:1546940–1,547,026	UGACAGAAGAGAGUGAGCAC
Lan-miR-156a-3		Scaffold_166_14:63382–63,466	UUGACAGAAGAGAGUGAGCAC
Lan-miR-156b		Scaffold_33:2890279–2,890,538	UUGACAGAAGAGAGAGAGCAC
Lan-miR-157	miR157	Scaffold_347:76018–76,134	UUGACAGAAGAUAGAGAGCAC
Lan-miR-159-1	mir159	Scaffold_42_76:293925–294,101	UUUGGAUUGAAGGGAGCUCU
Lan-miR-159-2		Scaffold_122_37:106935–107,104	UUUGGAUUGAAGGGAGCUCU
Lan-miR-159-3		Scaffold_416:58038–58,209	UUUGGAUUGAAGGGAGCUCU
Lan-miR-162-1	miR162	Scaffold_24_1:803070–803,147	UCGAUAAACCUCUGCAUCCAG
Lan-miR-162-2		Scaffold_56:1749922–1,750,000	UCGAUAAACCUCUGCAUCCAG
Lan-miR-164-1	miR164	Scaffold_3_382:600977–601,066	UGGAGAAGCAGGGCACGUGCA
Lan-miR-164-2		Scaffold_40_1:135439–135,578	UGGAGAAGCAGGGCACGUGCA
Lan-miR-164-3		Scaffold_43:1566397–1,566,711	UGGAGAAGCAGGGCACGUGCA
Lan-miR-166-1	miR166	Scaffold_14_1:1049417–1,049,545	UCGGACCAGGCUUCAUUCCCC
Lan-miR-166-2		Scaffold_75_81:26353–26,441	UCGGACCAGGCUUCAUUCCCC
Lan-miR-166-3		Scaffold_75_81:26549–26,680	UCGGACCAGGCUUCAUUCCCC
Lan-miR-166-4		Scaffold_84:15176–15,316	UCGGACCAGGCUUCAUUCCCC
Lan-miR-166-5		Scaffold_112_9:89703–89,800	UCGGACCAGGCUUCAUUCCCC
Lan-miR-166-6		Scaffold_205:416775–417,037	UCGGACCAGGCUUCAUUCCCC
Lan-miR-166-7		Scaffold_211:92403–92,516	UCGGACCAGGCUUCAUUCCCC
Lan-miR-166-8		Scaffold_214:184166–184,347	UCGGACCAGGCUUCAUUCCCC
Lan-miR-166-9		Scaffold_384:367108–367,321	UCGGACCAGGCUUCAUUCCCC
Lan-miR-167a	miR167	Scaffold_13_17:116311–116,381	AUUAGAUCAUGUGGCAGUUUCACC
Lan-miR-167b		Scaffold_28_51:550481–550,563	UGAAGCUGCCAGCAUGAUCUGA
Lan-miR-167c		Scaffold_562:8600–8671	UGAAGCUGCCAGCAUGAUCUUA
Lan-miR-168	miR168	Scaffold_53:393759–393,871	UCGCUUGGUGCAGGUCGGGAA
Lan-miR-319	miR319	Scaffold_328:212542–212,615	UUUGGACUGAAGGGAGCUCCU
Lan-miR-390-1	miR390	Scaffold_94_15:916791–916,877	AAGCUCAGGAGGGAUAGCGCC
Lan-miR-390-2		Scaffold_232:453780–453,886	AAGCUCAGGAGGGAUAGCGCC
Lan-miR-393	mirR393	Scaffold_73:439631–439,729	AUCAUGCUAUCCCUUUGGAUU
Lan-miR-396a-1	miR396	Scaffold_24_1:1251989–1,252,099	UUCCACAGCUUUCUUGAACUG
Lan-miR-396a-2		Scaffold_28_10:16613–16,706	UUCCACAGCUUUCUUGAACUG
Lan-miR-396b-1		Scaffold_24_1:1243559–1,243,896	UUCCACAGCUUUCUUGAACUU
Lan-miR-396b-2		Scaffold_56:1360367–1,360,458	UUCCACAGCUUUCUUGAACUU
Lan-miR-396b-3		Scaffold_154_72:424217–424,325	UUCCACAGCUUUCUUGAACUU
Lan-miR-398	miR398	Scaffold_73:1163532–1,163,624	CGUGUUCUCAGGUCGCCCCUG
Lan-miR-399a	miR399	Scaffold_27_10:3938–4026	GGGCACAUCUCUUUUGGCAAU
Lan-miR-399b		Scaffold_13_17:180470–180,547	UGCCAAAGGAGAGUUGCCCUG
Lan-miR-399c		Scaffold_40_1:2272903–2,273,002	UGCCAAAGGAGAUUUGUCCUG
Lan-miR-403	miR403	Scaffold_115_1:411013–411,215	UUAGAUUCACGCACAAACUUG
NovmiR01	–	Scaffold_2_412:1857797–1,857,926	ACAACGUAUGAGACAAGAUCU
NovmiR02	–	Scaffold_54_50:762283–762,384	CCACUUCUUUCAAACAGGCCC
NovmiR03	–	Scaffold_60_104:6844–7014	AAAGAAGUGUAGGACAACAGAUGU
NovmiR04	–	Scaffold_66_204:47747–47,849	UUGCCUUAUUGAGUUUGAGUUG
NovmiR05	–	Scaffold_75_88:63943–64,304	UCACUCCAACUUUGACCUUCU
NovmiR06	–	Scaffold_95_88:201776–201,867	UUCGUUUGUGUGCAGACUCUGC
NovmiR07	–	Scaffold_117:636044–636,156	AGCGUAAACUGAUUAACCAAGGGU
NovmiR08	–	Scaffold_154_72:75651–75,880	AGAGGUGUAUGGCACAAGAGA
NovmiR09	–	Scaffold_221_1:266662–266,818	UAGGUCAAAAAUGGAGUGAUG
NovmiR10	–	Scaffold_221_1:662328–662,414	UGUCGCAGGAGUGAUAGUACC
NovmiR11	–	Scaffold_256:144745–144,827	UCCGUGUAUUUGUACAAUAUC
NovmiR12	–	Scaffold_460:184017–184,178	CUGCAUGUUGUCUUUGGCCACC
NovmiR13	–	Scaffold_162_2:128835–128,917	UGGACAACGACGAUCUUCGCC

Fourty three lupin miRNAs belong to a total of 16 known miRNA families and 13 novel lupin miRNAs were predicted. The genomic locations of the predicted miRNA loci are given as well as the mature miRNA sequence

We identified seven highly conserved miRNA plant families (miRNA 156, 159/319, 165/166, 167, 390, 396) being present across Embryophyta. Furthermore, we identified seven miRNA families (miRNAs 162, 164, 168, 393, 398, 399, 403) that are also considered highly conserved among angiosperms. We also found two miRNA families (miRNAs 1511, 1507) that are less conserved and considered core Rosids miRNAs (which include Fabaceae) [41, 70]. Multiple distinct genomic loci were predicted to produce each of the conserved miRNA families. For example, nine genomic loci were predicted to produce miR166, whereas the newly evolved legume specific miRNA families (miR1507, miR1511) were produced by one or two genomic loci. All the novel predicted miRNAs only have one genomic locus as their origin.

**Fig. 3:**
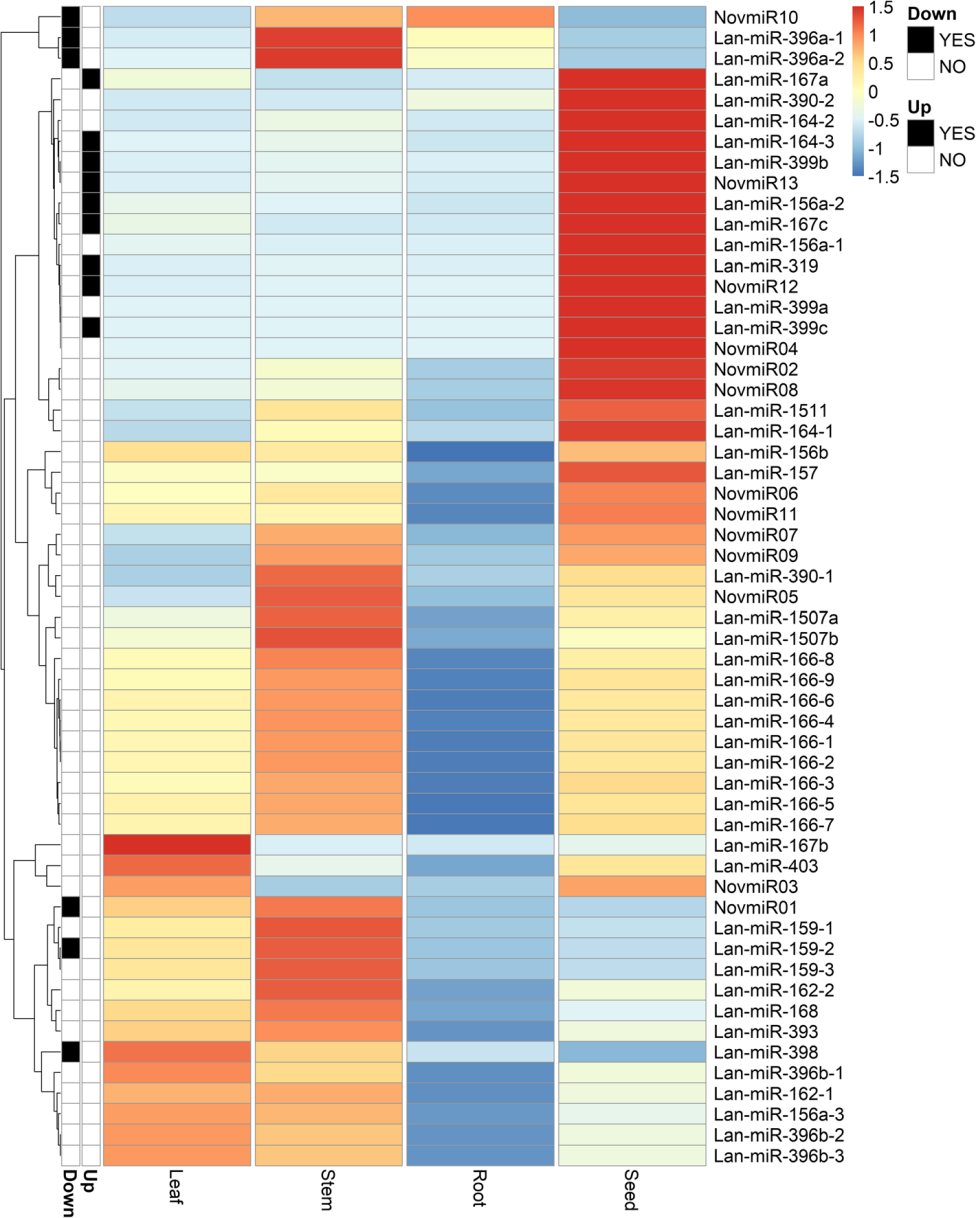
A heatmap showing the relative expression of predicted miRNAs in reads per million (RPM). Seed expression is shown as the average RPM of the five seed stages. Hierarchical clustering shows the main patterns of expression. Differential expression analysis using edgeR on the three vegetative samples (leaf, stem, root) versus the five seed stages was used to assess up- or down-regulation in seeds

miRNAs have a pivotal role during seed development as part of an intricate regulatory network controlling complex spatio-temporal transcriptional expression patterns that enable stage-specific developmental processes to occur [71, 72]. As such, we examined the expression profiles from the small RNA sequencing of the predicted miRNAs across five seed development stages, which encompassed embryogenesis, maturation and the onset of seed dormancy (Fig. 3). We detected highest relative expression of members of the miR167, miR390, miR164, miR399, miR156/157, miR1511 and mir319 families in seed (Fig. 3), as well as of seven novel predicted miRNAs (NovmiR13, NovmiR12, NovmiR04, NovmiR02, NovmiR08, NovmiR06, NovmiR11). We then performed a differential expression analysis comparing the combined seed stages versus the combined vegetative stages (leaf, stem, root) and found that Lan-miR-156a-2, Lan-miR-164-3, Lan-miR-167a/c, Lan-miR-319, Lan-miR-399b/c, NovmiR12 and NovmiR13 are up-regulated in seeds.

**Fig. 4:**
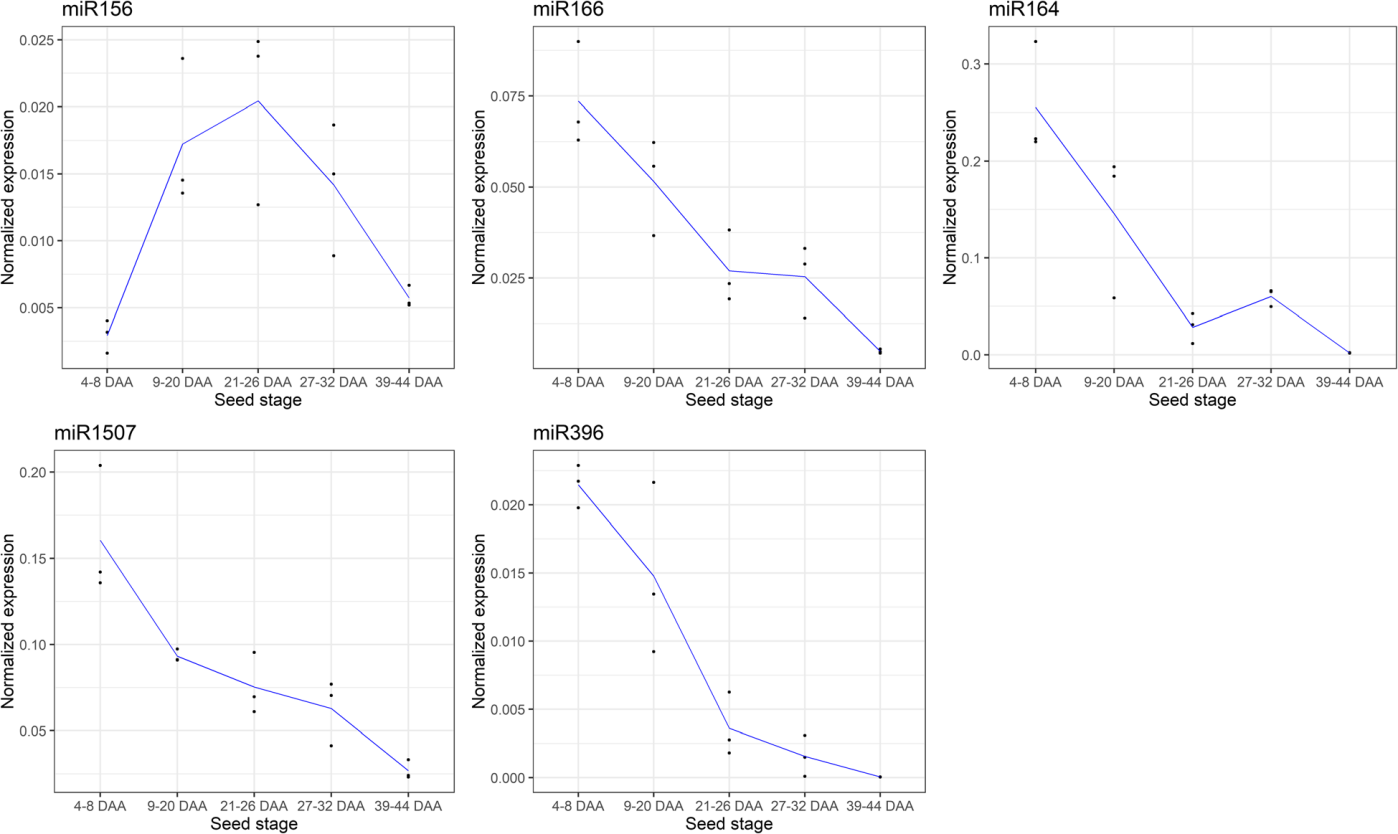
qPCR analysis of miR156/157, miR166, miR164, and miR396 expression levels in five seed developmental stages (4–8 DAA, 9–20 DAA, 21–26 DAA, 27–32 DAA, and 39–44 DAA). Expression was normalised to a random small RNA spiked into the samples prior to RNA extraction. Data is drawn as scatter plot with a line indicating the mean of the three replicates

Potential roles in seed development, dormancy, and germination have been suggested for miR156, miR160, miR169, and miR396 [73]. We confirmed the expression of miR156, miR396 as well as miR166, miR164 and miR1507 using quantitative RT-PCR during narrow-leafed lupin seed development (Fig. 4). This confirmed their expression during seed development and revealed distinct expression profiles during the different stages of seed development. For example, whilst miR166, miR164, miR1507 and miR396 are expressed highly during the early seed stages, miR156 is expressed highly in mature seeds.

### Identification of novel miRNA sequence within the small RNA datasets

We also found evidence for a novel miRNA complement within narrow-leafed lupin, namely 13 miRNAs predicted by ShortStack that do not share significant sequence similarity with known miRNA families deposited on miRBase (Table 1). Of the 13 novel miRNAs, we found two to be up-regulated in seed (NovmiR12 and NovmiR13) (Fig. 3). The novel miRNA sequences were predominately 21 nt (61.5%) or 22 nt (23.1%) in length, with a higher proportion of 22 nt sequences comparatively to the conserved miRNAs (21% are 21 nt in length, 9.3% are 22 nt in length). We also observed a 5′ uridine nucleotide bias in 53.9% of the novel miRNA sequences.

An additional class of DCL3 dependent 24 nt long miRNAs, which direct DNA methylation at target loci and also at their own loci through association with AGO4, has been described in rice [74, 75]. One of the conserved miRNAs and two of the novel predicted miRNAs were 24 nt in length that could potentially be classified as novel long miRNAs. The mature miRNA sequences all contained a 5′ adenine nucleotide.

### Distribution of the miRNA sequences in the narrow-leafed lupin genome

**Fig. 5:**
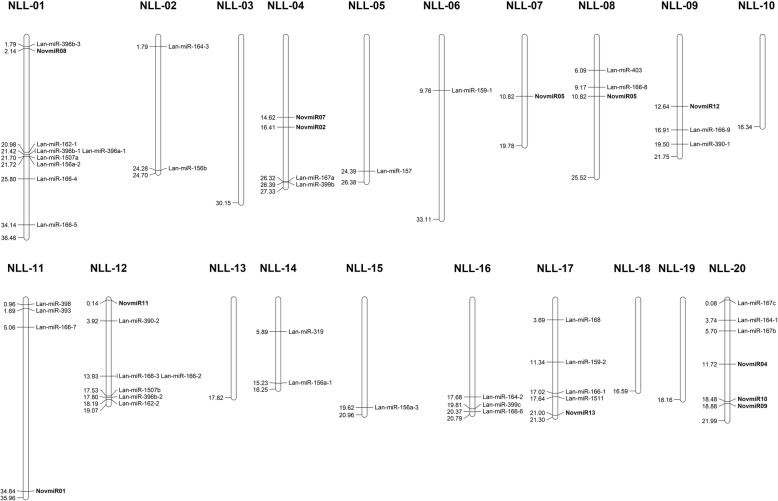
Distribution of miRNAs across the 20 narrow-leafed lupin chromosomes. The position of each miRNA has been shown relative to the pseudochromosome coordinates of the narrow-leafed lupin assembly [12] with the novel miRNAs distinguished in bold from the conserved miRNAs. The value on the left of the pseudochromosome represent the physical location in Mb of a given miRNA presented on the right of each pseudochromosome

MiRNA loci are usually located in intergenic regions within the genomes of plants [39]. To determine whether a similar distribution is observed within narrow-leafed lupin, we analysed the genomic location of both the conserved and novel miRNA candidates. The genomic coordinates (e.g. scaffold coordinates) of the miRNAs were used to assign the miRNAs to a chromosome to determine the distribution of the miRNAs across the 20 chromosomes of narrow-leafed lupin (Fig. 5). The miRNA sequences were dispersed through the lupin genome, although no miRNAs were identified on chromosomes NLL-03, NLL-10, NLL-13, NLL-18 and NLL-19. Four of the 56 miRNA identified were not on scaffolds in the pseudochromosome assembly including one novel miRNA (NovmiR03). NLL-01 harbours the largest number of miRNAs, nine including one novel miRNA (NovmiR08). Novel miRNAs were distributed across nine different chromosomes with NLL-20 harbouring three novel miRNAs (NovmiR04, NovmiR09 and NovmiR10). Members of conserved miRNAs were distributed across the chromosomes rather than clustered together, which is consistent with observations in other plant species [40]. However, three of the five Lan-miR-396 family members reside on NLL-01, whereas one resides on NLL-12, with the other on a scaffold yet to be assigned to a pseudochromosome.

The majority of the conserved miRNA sequences (93%) were located within the intergenic regions (Additional file 5: Table S3). The remaining 7% of sequences were located within the UTR regions of annotated transcripts, with no sequences located within the intron/exon regions. In contrast, only 46.2% of the novel miRNA sequences were located within intergenic regions, 30.8% were located within introns and the remaining 23.1% located within the UTRs or exons of annotated transcripts (Additional file 5: Table S3). This suggests that lupin harbours both conserved miRNAs that are typically found in intergenic regions in plants as well as evolutionary recent miRNAs that appear to originate from introns, exons or UTRs.

### miRNA target identification

Putative miRNA targets were identified using the psRNATarget software and narrow-leafed lupin transcriptome datasets previously generated by our laboratory. Using stringent parameters (expectation cut-off score of 2.5), 49 putative unique targets for the conserved miRNAs were identified (Additional file 6: Table S4). Whilst some bona fide targets may not be identified by using stringent parameters, this was considered to be preferable than the inclusion of large numbers of false positive targets within our datasets. A large proportion of genes encoding for transcription factors (40.4%) were identified as putative miRNA targets. Analysis of the InterPro terms revealed the presence of several different classes of transcription factors within our datasets including; GROWTH-REGULATING FACTOR (GRF) transcription factors, SBP-box transcription factors, MYB transcription factors and Zinc finger domain proteins (Additional file 6: Table S4). The remaining targets encoded proteins involved in various other cellular processes.

We identified 20 unique genes that were putatively targeted by novel miRNAs (Additional file 7: Table S5). We did not identify targets for five of the novel miRNAs (NovmiR03, NovmiR06, NovmiR07, NovmiR09 and NovmiR12). Only 20% of the identified targets for the novel miRNAs encoded transcription factors (MYB and BIM1 transcription factors and B3 domain-containing proteins). The remaining targets encode proteins with a diverse array of functions. Among these targets we found proteins annotated as a molybdate transporter 1, a calcium-transporting ATPase 8, a TMV resistance protein N, a lysine-specific demethylase JMJ16 and a nudix hydrolase protein (Additional file 7: Table S5). The functional diversity of proteins targeted by linage specific miRNAs has also been reported in other plant species [76, 77].

### miRNA target validation using 5′ RLM-RACE

**Fig. 6:**
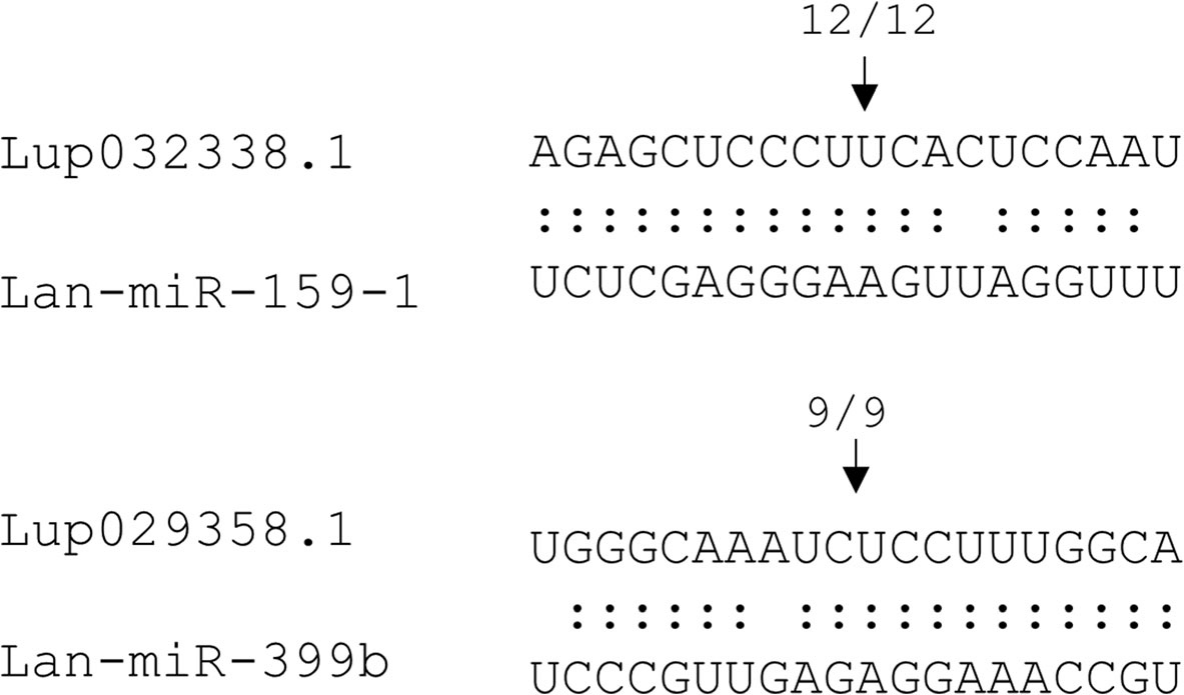
Mapping of the cleavage sites for two conserved miRNAs targets using modified 5′ RLM- RACE. *Lup029358.1* encodes a putative Ubiquitin-conjugating E2 enzyme, involved in maintenance of phosphate homeostasis in other plant species [78] and *Lup032338.1* encodes a putative MYB transcription factor

miRNA-mediated target cleavage occurs precisely between the 10th and 11th nucleotide upstream of the 5′ end of the miRNA sequence, and as such the cleavage sites can be validated using 5′ RLM-RACE [58, 59]. Using this approach, we attempted to validate the targets for two conserved miRNAs (Lan-miR-399, Lan-miR-159) and were successful in each case. Thus, we validated miR399 mediated cleavage of *Lup029358.1*, a putative Ubiquitin-conjugating E2 enzyme, involved in maintenance of phosphate homeostasis in other plant species [78] (Fig. 6). We also validated the cleavage of *Lup032338.1*, a putative MYB transcription factor targeted by miR159 (Fig. 6).

## Discussion

Narrow-leafed lupin, an emerging crop species, has only recently been domesticated and the germplasm still contains several undesirable phenotypic traits within the seed grain [79]. MiRNAs are key regulators of numerous plant developmental processes and as such represent a potential mechanism to manipulate and improve agronomic traits such as yield and nutritional quality of the seed. Here we have sequenced the small RNA population from several different seed stages of narrrow-leafed lupin as well as the foliar tissue with the aim of identifying miRNAs that may contribute to seed development, and thus represent potential targets for miRNA-based plant improvement.

### Dicer-like and Argonaute orthologs within the lupin genome

Initially we were interested in examining the genetic composition of important components of the small RNA biogenesis machinery within the narrow-leafed lupin genome, particularly as the Genistoid clade is quite phylogenetically distant from other well characterised legumes [12, 14]. We identified seven *Dicer-like* and 16 *Argonaute-like* genes in the narrow-leafed lupin genome assembly that shared a high level of homology with their legume orthologs. The number of *AGO* genes in other legumes is highly variable, with *M. truncatula* containing 12 *AGO* genes, *G. max* (soybean) containing 20 *AGO* genes while only 9 *AGO* genes have been identified in the *L. japonicus* genome [42]. The number of *AGO* genes in other more distantly related plant species is also highly variable, with *A. thaliana* containing 10 *AGO* genes [80] and rice containing 19 *AGO* genes [81, 82]. Genome duplication events may underlie the rapid expansion of the *AGO* gene family during plant evolution, facilitating the functional diversification of the AGO proteins, and contributing to the complexity of the small RNA pathways in plants [81, 83]. A recent whole genome triplication event within narrow-leafed lupin [12, 14] and a whole genome duplication event within soybean [84], could potentially explain the increase in *AGO* gene copies within both these species, comparatively to other legumes. Interestingly, the number of *DCL* genes were comparable between the legume species examined, with both *M. truncatula* [60] and *L. japonicus* [42] containing 6 *DCL* genes, and *G. max* containing 7 *DCL* genes [42, 60], similar to what was found for narrow-leafed lupin. It is possible that functional constraints such as gene dosage effects, may have contributed to gene loss events following the whole genome duplication events in both narrow-leafed lupin and soybean. Whether the AGO and the DCL proteins identified in narrow-leafed lupin and other legume species have diversified and evolved additional novel functional roles to those described in other plant species, remains to be elucidated. Experimental analysis of mutants, generated through mutagenesis screens or via emerging technologies such as CRISPR/CAS9, is required to understand the roles of these proteins in the miRNA and siRNA pathways. Nevertheless, our analyses suggest that the Genistoid legume narrow-leafed lupin possesses the required genes for small RNA biogenesis with the identification of 7 *Dicer-like* and 16 *Argonaute-like* genes.

### Characteristics of the small RNA population throughout seed development

To examine the involvement of small RNAs during seed development, we undertook a comprehensive analysis of the narrow-leafed lupin small RNA populations using Illumina Small RNA-Seq technology. At each seed developmental stage examined, the small RNA populations were mainly comprised of 24 nt sequences, with 21 nt sequences also abundant within the datasets. Similar compositions have been reported within a variety of tissues from other legumes such as *M. truncatula* [85] and chickpea [76], though within the developing soybean seed, each of the 21, 22 and 24 nt small RNA populations were all highly abundant [86]. The enrichment of the 24 nt small RNA population within narrow-leafed lupin seed, is consistent with heterochromatin siRNAs, derived from transposons and repetitive genomic elements, being a major class of small RNAs within plants [17]. Interestingly, the 24 nt small RNA population was less abundant in the foliar tissue and root libraries as well as the late seed stage 39–44 DAA library (34.2, 31 and 51.4% of total read counts respectively) compared to libraries generated from the earlier seed stages, where this population represented 64–71% of the total read counts. Dynamic changes in the 24 nt small RNA population during seed development has also been reported within *B. napus* [87]. Concomitant with the enrichment of the 24 nt small RNA population within narrow-leafed lupin, we observed high expression of the *AGO4* orthologs within the seed. AGO4 associates with 24 nt small RNAs, mediating DNA methylation via the RNA-directed DNA methylation (RdDM) pathway in other plants species [62]. Thus, these observations suggest an important role for AGO4 and associated 24 nt small RNAs in the RNA-directed DNA methylation (RdDM) pathway during seed development.

### A large complement of conserved miRNAs were identified in narrow-leafed lupin throughout seed development

To further elucidate the molecular mechanisms underlying narrow-leafed lupin seed development we identified the miRNA complement expressed within the seed. We identified a total of 43 conserved miRNA sequences, which were classified into 16 known miRNA families. Multiple sequence variants were identified within a number of the highly conserved miRNA families, many of which mapped to multiple genomic loci, which may be reflective of gene expansion events that have occurred throughout lupin evolution consistent with what has been observed in Arabidopsis [77]. Whilst there was some size heterogeneity, the conserved miRNAs were predominately 21 nt in length (76.7%) with lower proportions of 20 nt and 22 nt sequences also present. We only detected one 24 nt variant of the conserved miRNA sequences, which has also been described within other plant species [88]. In some cases, distinct size variants originating from the same precursor were observed. These variants exhibited sequence heterogeneity at the termini regions possibly due to imprecise DCL processing or through post-transcriptional modifications [89], though end-degradation cannot be excluded. Large repertoires of miRNA sequence variants have been reported within other plants species [90], with differences in target selection, stability or AGO loading efficiency potentially altering biological activity [89, 91]. Although the sequence variants identified here were for the most part predicted to have overlapping targets, we did observe differences in the expression profiles for some variants throughout seed development, which may be indicative of differing biological activities. Tissue specific and developmental specific expression of miRNAs has also been reported in other plant species [59, 92].

### Transcription factors and hormone signalling pathways are targeted by conserved miRNAs throughout seed development

The miRNA-regulatory networks controlling seed developmental processes such as embryonic patterning, organ polarity and apical meristem formation, are evolutionarily conserved across a wide range of species [93]. Accordingly, we detected a number of miRNAs (miR156, miR164, miR166 and miR396) with previously characterised roles in seed development in other species, many of which exert their regulatory effects through the repression of transcription factors. In *A. thaliana,* miR156 controls the morphogenesis-maturation phase transition during embryogenesis through repression of SBP-Box transcription factors [19]. We also detected expression of the miR164 family during lupin seed development, consistent with the role of miR164 in embryonic pattern formation in *A. thaliana* via the regulatory control of NAC (No Apical Meristem) domain containing transcription factors [23, 94]. The miR166 family was also detected during narrow-leafed lupin seed development. In *A. thaliana,* miR166 targets Class III HD-Zip transcription factors regulating several biological processes during embryogenesis, including lateral organ polarity and shoot apical meristem formation [95–97]. As each of these miRNAs were abundant throughout lupin seed development, and were predicted to target similar genes as those described in other plant species, they are likely to represent key regulators of narrow-leafed lupin seed development.

### Novel narrow-leafed lupin–specific miRNA complement expressed throughout narrow-leafed lupin seed development

Newly evolved miRNAs, once integrated into regulatory networks, can contribute to morphological and physiological diversity within the plant, and thus represent avenues for agronomic trait improvement. As such, we were interested in examining the novel narrow-leafed lupin–specific miRNA complement throughout narrow-leafed lupin seed development. We identified a total of 13 narrow-leafed lupin specific novel miRNAs in our datasets that were between 21 and 24 nt in length. Novel miRNAs were represented by a single sequence variant and transcribed from a single genomic location, consistent with observations in other plant species [40, 41]. According to the current model of plant miRNA evolution, newly emerged miRNA have a high turnover rate, with many miRNA being lost soon after their formation [41]. These ‘young’ species-specific miRNA genes are often expressed at low levels compared to the highly conserved miRNAs and their regulatory effect and range of targets is expected to be lower. Further investigation of these ‘young’ narrow-leafed lupin-specific miRNA may be warranted, as one or more may play a role in narrow-leafed lupin development.

We also identified three seemingly bona fide 24 nt long miRNAs with narrow-leafed lupin. In *A. thaliana,* conserved miRNAs are primarily processed by DCL1 to produce 21–22 nt mature miRNAs, with a small number of DCL1 dependent 24 nt miRNAs also described [41]. Here size variations in the mature miRNA sequences have been attributed to characteristics of the fold-back precursor structure, specifically the presence of asymmetric bulged nucleotides within the miRNA duplex [98, 99]. An additional class of long 24 nt miRNAs, processed instead by DCL3, and which mediate DNA methylation at target loci through association with AGO4 clade proteins, has also been described in rice and *A. thaliana* [74, 75]. Here the differences in DCL usage are hypothesised to relate to the fold-back structure of the miRNA precursor [88]. Analysis of the small RNA populations within *DCL1*, *DCL3* and *RDR*2 lupin mutants, will provide additional insight into the biogenesis and the function of the putative 24 nt long miRNAs during narrow-leafed lupin seed development.

## Conclusion

Here we identified *AGO* and *DCL* genes important for the biogenesis of miRNAs in narrow-leafed lupin and highly abundant miRNAs in different developmental stages of the narrow-leafed lupin seed. These miRNAs predominantly targeted transcription factors, some of which have known roles in gene regulation during seed development in other crops. The data presented here on conserved and narrow-leafed lupin specific novel miRNAs during seed development and maturation, adds to the toolbox of genetic and genomic resources for narrow-leafed lupin, offering opportunities to improve the quality of lupin grain, which is increasingly being used for human consumption and as an attractive non-GMO, gluten free, high protein alternative to soybean.

## Additional files

Additional file 1: ShortStack annotation of the predicted miRNA loci. (ZIP 41 kb)

Additional file 2: **Table S1.** qPCR and 5′ RLM-RACE primer sequences. (XLSX 14 kb)

Additional file 3: **Table S2.** List of *AGO*- and *DCL-like* genes identified in the narrow-leafed lupin cultivar Tanjil reference genome assembly. (XLSX 10 kb)

Additional file 4: **Figure S1.** (A) narrow-leafed lupin *AGO-*like and (B) narrow-leafed lupin *DCL*-like gene expression profiles across different tissue types in RNASeq datasets described in [11]. (TIF 1054 kb)

Additional file 5: **Table S3.** List of the genomic locations of conserved and novel miRNAs. (XLSX 14 kb)

Additional file 6: **Table S4.** List of the target annotations for the conserved miRNAs. (XLSX 13 kb)

Additional file 7: **Table S5.** List of the target annotations for the novel miRNAs. (XLSX 10 kb)

## Abbreviations

DAA: days after anthesis
miRNAs: micro RNAs narrow-leafed lupin, narrow-leafed lupin
siRNAs: small interfering RNAs
