# Supplementary figures and images for: Identification and profiling of narrow-leafed lupin (Lupinus angustifolius) microRNAs during seed development
Source: BMC Genomics. 2019 Feb 14;20:135. doi: 10.1186/s12864-019-5521-8 (PMC6376761; doi:10.1186/s12864-019-5521-8)

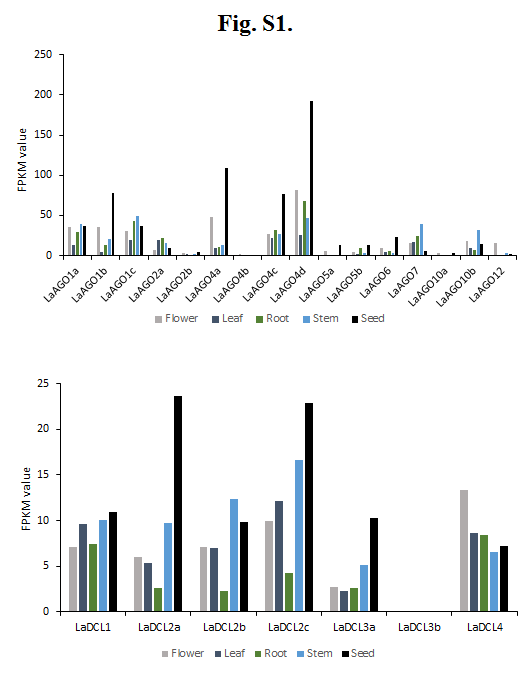

Supplement: Supplementary file 4 — Figure S1. (A) narrow-leafed lupin AGO-like and (B) narrow-leafed lupin DCL-like gene expression profiles across different tissue types in RNASeq datasets described in [11]. (TIF 1054 kb) [file 12864_2019_5521_MOESM4_ESM.tif]
